# Particle‐Assisted Optoelectronic Tweezers for Manipulating Single Cells and Microparticles

**DOI:** 10.1002/advs.202501032

**Published:** 2025-05-05

**Authors:** Ao Wang, Shuzhang Liang, Caiding Ni, Yongyi Jia, Kangjie Wu, Wenyan Niu, Shunxiao Huang, Kaiyi Peng, Chutian Wang, Yingjian Guo, Zhijun Zhao, Lingze Zhang, Mingjie Liu, Lin Feng

**Affiliations:** ^1^ School of Mechanical Engineering and Automation Beihang University Beijing 100191 China; ^2^ Department of Mechanical Engineering The University of Tokyo Tokyo 113‐8656 Japan; ^3^ Department of Automation Tsinghua University Beijing 100084 China; ^4^ Central Laboratory Peking University First Hospital Ningxia Women and Children's Hospital Yichuan 750001 China; ^5^ School of Chemistry Beihang University Beijing 100191 China; ^6^ Beijing Advanced Innovation Center for Biomedical Engineering Beihang University Beijing 100191 China

**Keywords:** indirect manipulation, optoelectronic tweezers, particle‐induced dielectrophoresis, single‐cell manipulation

## Abstract

Manipulation of single cells or particles is crucial in the biomedical field. However, precisely and rapidly manipulating single cells without damaging them is a significant challenge. In this study, a novel strategy for indirect manipulation of cells and microparticles that can satisfy these requirements via a combination of particle‐induced dielectrophoretic forces (PiDEP) and optoelectronic tweezers (OET) is developed. This strategy is based primarily on the principle that particles experiencing the same dielectrophoretic forces tend to repel each other, whereas those experiencing different forces are attracted to each other. During the manipulation, Ag‐SiO_2_ microparticles controlled by the OET act as intermediaries for manipulating other particles or cells through dielectrophoretic forces. Thus, the manipulation range of the OET can be expanded by two to three times its original size, and the manipulation speed can be significantly increased while maintaining its precision. Furthermore, the results indicate that the proposed method can effectively reduce cell damage to one‐third of that caused by traditional OET. This study demonstrates the significant potential of particle‐assisted OET for single‐cell manipulation and offers an effective strategy for manipulating cells and microparticles.

## Graphical abstract

This work introduces a particle‐assisted optoelectronic tweezers (OET) technique that uses Ag‐SiO_2_ microparticles as intermediaries to manipulate cells and microparticles via particle‐induced dielectrophoresis. The method extends the OET manipulation range while reducing cell damage. Its compatibility with microfluidic systems enables precise control in complex environments, thereby advancing applications in biomedical research and microrobotics.

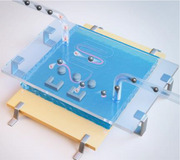

## 1 Introduction

Cells are the basic structural and functional units of organisms. Even within the identical environment, there exists significant heterogeneity among cells in a population.^[^ ^1^, ^2^ ^]^ Studying cellular heterogeneity is crucial in fields such as antibody drug discovery,^[^ ^3^ ^]^ single‐cell genomics research,^[^ ^4^ ^]^ and regenerative medicine.^[^ ^5^ ^]^ To address this need, researchers have developed diverse field‐based control technologies to manipulate and screen individual cells, including optical,^[^ ^6^ ^]^ acoustic,^[^ ^7^ ^]^ magnetic,^[^ ^8^, ^9^ ^]^ and electric fields.^[^ ^10^, ^11^ ^]^ Among these, optical field manipulation has attracted particular attention in the field of single‐cell control due to its high precision and operational flexibility. Numerous optical field–based techniques have since been developed, such as optical tweezers,^[^ ^12^, ^13^, ^14^ ^]^ optothermal tweezers,^[^ ^15^ ^]^ photopyroelectric tweezers,^[^ ^16^ ^]^ optically induced electrostatics,^[^ ^17^ ^]^ and optoelectronic tweezers (OET).^[^ ^18^ ^]^

OET, also termed optically induced dielectrophoresis (ODEP), stand out as a noncontact parallel manipulation technology that differ from numerous single‐cell manipulation methods. A wide range of objects can be manipulated by OET, including biological entities (various cells,^[^ ^19^, ^20^, ^21^, ^22^, ^23^, ^24^, ^25^ ^]^ bacteria,^[^ ^26^, ^27^ ^]^ and microalgae^[^ ^28^, ^29^ ^]^) and nonbiological entities (various microspheres,^[^ ^30^, ^31^, ^32^, ^33^ ^]^ nanoparticles,^[^ ^34^, ^35^, ^36^ ^]^ hydrogel microstructures,^[^ ^37^, ^38^ ^]^ and metal microrobots^[^ ^39^ ^]^). Despite its versatility, OET has significant drawbacks. First, the electric field induced by OET can cause rapid cell lysis and death within a short period.^[^ ^40^, ^41^ ^]^ Second, cells are vulnerable to Joule heating under continuous and intense illumination.^[^ ^42^ ^]^ Consequently, the direct application of OET in single‐cell manipulation remains challenging.

Recent approaches have attempted to address these issues. For instance, we previously used polystyrene microspheres with negative DEP to capture, transfer, and release microorganisms with positive DEP responses.^[^ ^29^ ^]^ While this improved manipulation speed, it did not shield cells from light exposure. Similarly, Yossifon et al. used optical patterns for navigation by employing self‐propelling Janus particles to adsorb and transport 293T cells. However, this method failed to prevent the cells from being directly exposed to the light beams.^[^ ^43^ ^]^ Gear‐shaped microrobots offer indirect manipulation with reduced damage and improved transport speed,^[^ ^44^ ^]^ but their fabrication is complex, and their planar shape limits their use in microfluidic systems. In addition, cells may adhere to the microrobots during manipulation. Therefore, a simple and rapid single‐cell manipulation method that maintains cell viability has not yet been effectively developed.

**Figure 1:**
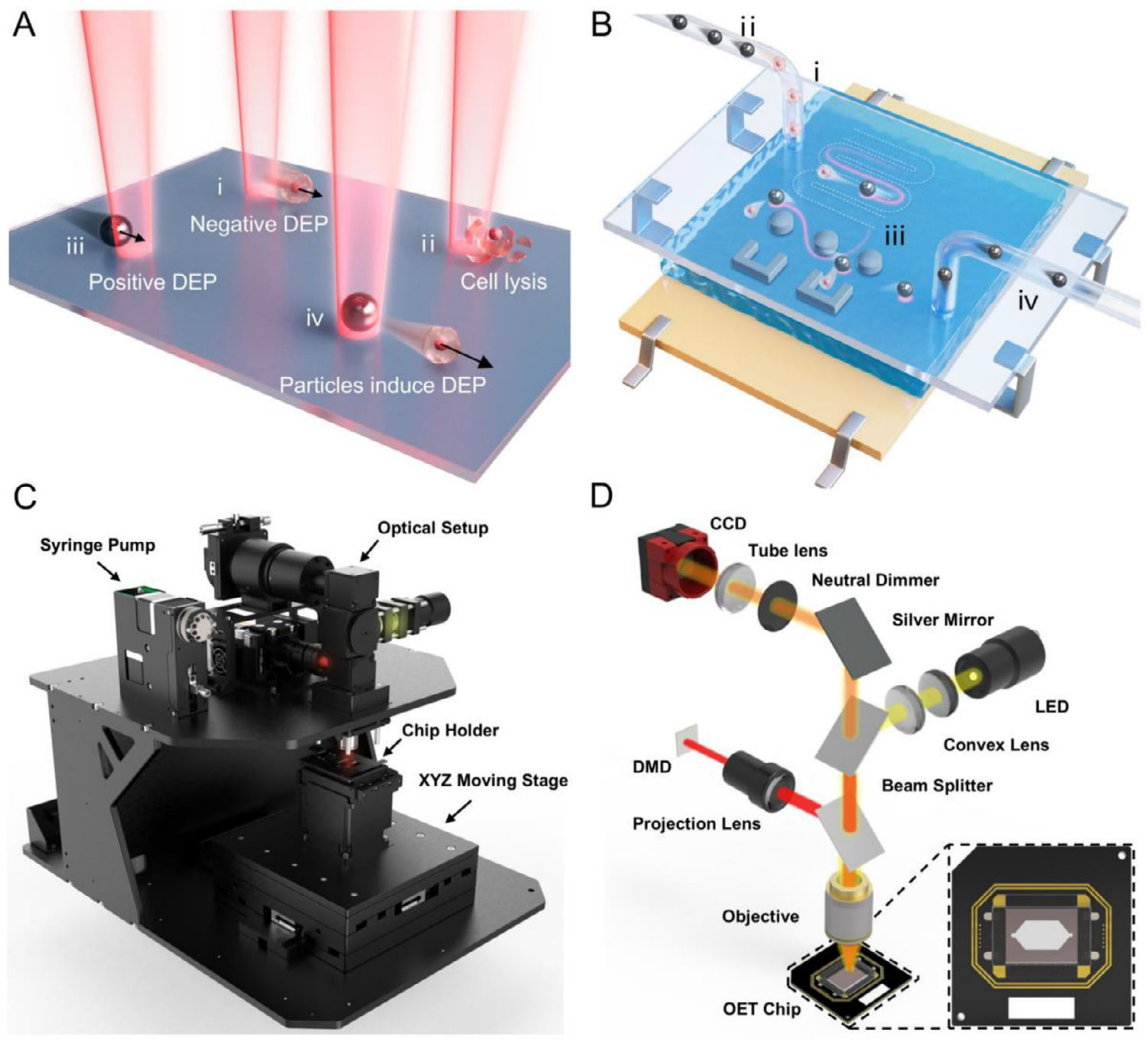
Schematic of the particle‐assisted OET. A) Effects of OET and PiDEP on particles. (i) Cells are pushed away from the light spot by the negative DEP of the OET. (ii) Direct manipulation of cells using OET may cause cell lysis and death. (iii) Ag‐SiO_2_ microspheres are attracted into the light spot by the positive DEP of the OET. (iv) The PiDEP between the Ag‐SiO_2_ microspheres and cells is used to drive cell movement. B) Process of utilizing the particle‐assisted OET to manipulate cells within a microfluidic chip to achieve complex movements. (i) Injection of cells. (ii) Injection of Ag‐SiO_2_ microspheres. (iii) Manipulation of cells for trajectory control, obstacle avoidance, and targeted release. (iv) Retrieval of Ag‐SiO_2_ microspheres. C) Overall composition of the OET system. D) Detailed schematic of the optical path in the OET system.

Inspired by the indirect particle manipulation strategies in optical tweezers,^[^ ^45^, ^46^, ^47^ ^]^ we introduced an intermediary particle into the OET system to enable noncontact, indirect manipulation of cells. When two particles approach each other under a direct current (DC) or alternating current (AC) electric field, the local electric field surrounding them is significantly altered, resulting in a dielectrophoretic force between the particles known as the particle‐induced dielectrophoretic force (PiDEP). We used OET to direct control Ag‐SiO_2_ microspheres, which served as “amplifiers” to drive nearby cells via repulsive PiDEP, significantly extending the manipulation range while preserving cell viability (**Figure** **1**A). As shown in Figure 1B, due to the size similarity between the intermediary microspheres and cells, this method supports precise manipulation in narrow and complex microchannels. The intermediary microspheres can be injected and retrieved via capillaries and are compatible with various microfluidic chips without sample contamination. Furthermore, this manipulation method was compatible with traditional OET experimental setups and optical systems (Figure 1C,D). We believe that particle‐assisted OET has promising potential applications in microparticle/cell manipulation, microsensing, and microrobotics.

## 2 Results

### 2.1 Direct Manipulation of Microspheres Using OET

**Figure 2:**
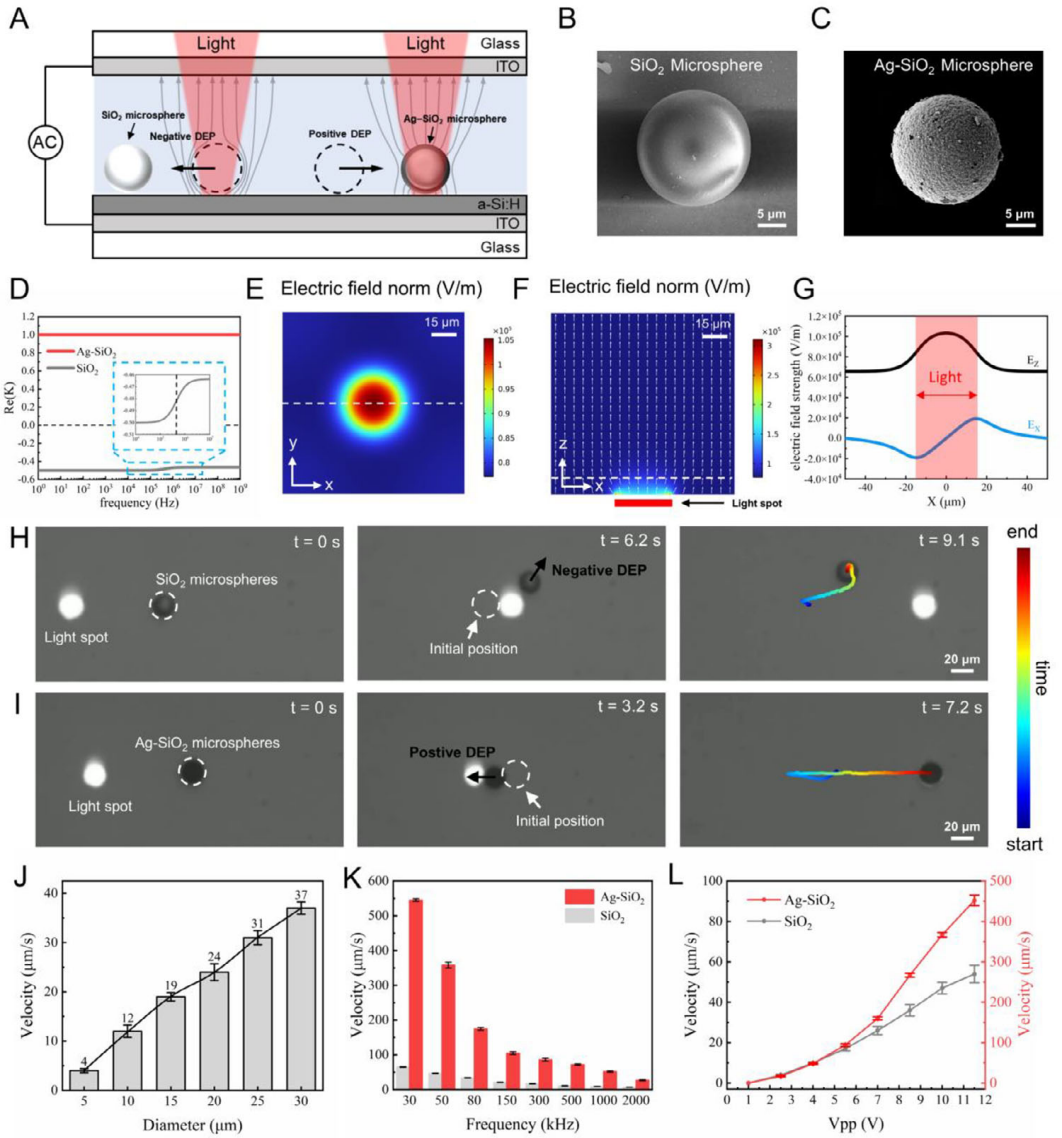
Principles and simulation analysis of direct manipulation of SiO_2_ and Ag‐SiO_2_ microspheres using OET. A) Schematic of the manipulation of SiO_2_ and Ag‐SiO_2_ microspheres by the ODEP force. B,C) Scanning electron microscope images of SiO_2_ and Ag‐SiO_2_ microspheres, respectively. D) Re(*K*) of SiO_2_ and Ag‐SiO_2_ microspheres as a function of the applied AC electric field frequency. E,F) Top and side views, respectively, of the electric field strength norm (blue for low, red for high) in the illuminated area of the OET chip (white arrows indicate the normalized electric field direction vectors). G) Electric field strength norm along the X‐ and Z‐directions (white dashed lines in panels (E) and (F), 10 µm from the substrate). Positive values of E_X_ and E_Z_ indicate the positive directions of the X‐ and Y‐axes. H,I) Time sequences of a circular light spot approaching SiO_2_ and Ag‐SiO_2_ microspheres, respectively, from left to right (the starting point is marked in blue and the ending point is marked in red). J) Manipulating velocity of SiO_2_ microspheres with diameters ranging from 5 to 30 µm for a conductivity of 2 × 10^−3^ S m^−1^, frequency of 50 kHz, and 10 Vpp. K) Manipulating velocity of SiO_2_ and Ag‐SiO_2_ microspheres with a 20‐µm diameter as a function of frequency for a 10‐Vpp sinusoidal voltage. L) Manipulating velocity of SiO_2_ and Ag‐SiO_2_ microspheres with a 20‐µm diameter as a function of Vpp for a 50‐kHz sinusoidal waveform.

**Figure** **2**A shows a schematic of microsphere manipulation in the OET chip. Briefly, the OET chip consisted of a photoconductive substrate, a microfluidic channel layer, and an indium tin oxide (ITO) conductive layer. The hydrogenated amorphous silicon (a‐Si:H) thin film on the photoconductive substrate was the most critical component. When a circular light pattern irradiated the a‐Si:H layer, the conductivity in the illuminated area increased by more than two orders of magnitude compared to that in the nonilluminated area.^[^ ^48^ ^]^ Under an AC electric field, the potential of the illuminated area was higher than that of other areas, which created a localized nonuniform electric field. At this point, the particles experiencing a negative DEP force were repelled from the light spot, whereas those experiencing a positive DEP force were attracted. The DEP force can be described by^[^ ^49^, ^50^ ^]^

(1) $$F_{DEP}=2\pi \varepsilon_{m}R^{3}Re\left(K\right)\nabla E^{2}$$

where *R* is the radius of the microsphere, ε_*m*_ is the dielectric constant of the medium, Re(*K*) is the real part of the Clausius–Mossotti (CM) factor, *K* represents the degree of polarization of the particle in the electric field, and *E* is the electric field strength. Equation (1) approximates the particle as a point dipole, which does not affect the electric field distribution in the original space.

The CM factor was the primary determinant of whether the particles experienced positive or negative DEP forces. Specifically, when Re(*K*) > 0, the particles experienced a positive DEP force, and when Re(*K*) < 0, the particles experienced a negative DEP force. SiO_2_ microspheres exhibit negative a DEP force in low‐conductivity media.^[^ ^51^ ^]^ To obtain particles that experienced a positive DEP force, we used electroless plating to uniformly deposit a metallic silver layer with a thickness of 100 nm on the SiO_2_ surface, resulting in Ag‐SiO_2_ microspheres (Figure 2B,C shows scanning electron microscope images of the SiO_2_ and Ag‐SiO_2_ microspheres). This metallic silver layer significantly altered the dielectric properties of the SiO_2_ microspheres.^[^ ^52^ ^]^ Based on our calculations (Note S1, Supporting Information), we obtained curves of Re(*K*) for the SiO_2_ and Ag‐SiO_2_ microspheres as a function of the AC electric field frequency (Figure 2D). The results showed that in a medium with a conductivity of 2 × 10^−3^ S m^−1^, Re(*K*) for the SiO_2_ microspheres was always less than zero, indicating that they experienced a negative DEP force. In contrast, Re(*K*) for the Ag‐SiO_2_ microspheres remained at one, indicating that they experienced a positive DEP force.

To investigate the electric field distribution, the photoconductivity and dark conductivity of the a‐Si:H film were measured (Figure S1 and Note S2, Supporting Information), and a finite element simulation model was subsequently developed based on the results (Figure S2 and Note S3, Supporting Information). The simulation results indicate that the electric field strength reaches its maximum at the edges of the light spot in the X‐direction, while in the Z‐direction, it peaks at the center (Figure 2E–G). Consequently, direct illumination of the SiO_2_ microsphere caused it to experience an upward force perpendicular to the substrate, propelling it out of the focal plane of the microscope. Only when the light spot approached the SiO_2_ microsphere from the side did it experience a horizontal DEP force (Figure 1H and Movie S1, Supporting Information). In contrast, when the light spot illuminated the area around the Ag‐SiO_2_ microsphere, it became tightly adsorbed onto the substrate and moved along with the light spot (Figure 1I and Movie S1, Supporting Information).

Next, we measured the maximum manipulation speeds and operational forces of SiO_2_ microspheres with various diameters. The results showed that as the particle diameter increased, the manipulation speed increased linearly (Figure 2J) and the DEP force increased quadratically (Figure S3A, Supporting Information). In addition, we observed that the manipulation speed of the SiO_2_ microspheres was significantly lower than that of the Ag‐SiO_2_ microspheres. As the AC frequency increased, the manipulation speed for both types of microspheres decreased significantly (Figure 1K). Furthermore, the experimental results showed that as the voltage increased, the manipulation speed of both types of microspheres improved (Figure 1L). In particular, for a rectangle‐wave AC power supply, the manipulation speed was approximately twice that for a sine‐wave power supply (Figure S3B,C, Supporting Information).

### 2.2 Interactions between Microspheres in the OET System

As the individual microparticles were manipulated, we observed electrostatic‐like interactions between the particles. This force arises when particles become polarized in an electric field, and this phenomenon is referred to as the particle‐induced dielectrophoretic force (PiDEP).^[^ ^53^, ^54^ ^]^ PiDEP can induce various behaviors in colloidal particles, cells, and other microparticles, such as dispersion,^[^ ^55^, ^56^ ^]^ aggregation,^[^ ^57^, ^58^, ^59^ ^]^ and chaining.^[^ ^58^ ^]^

**Figure 3:**
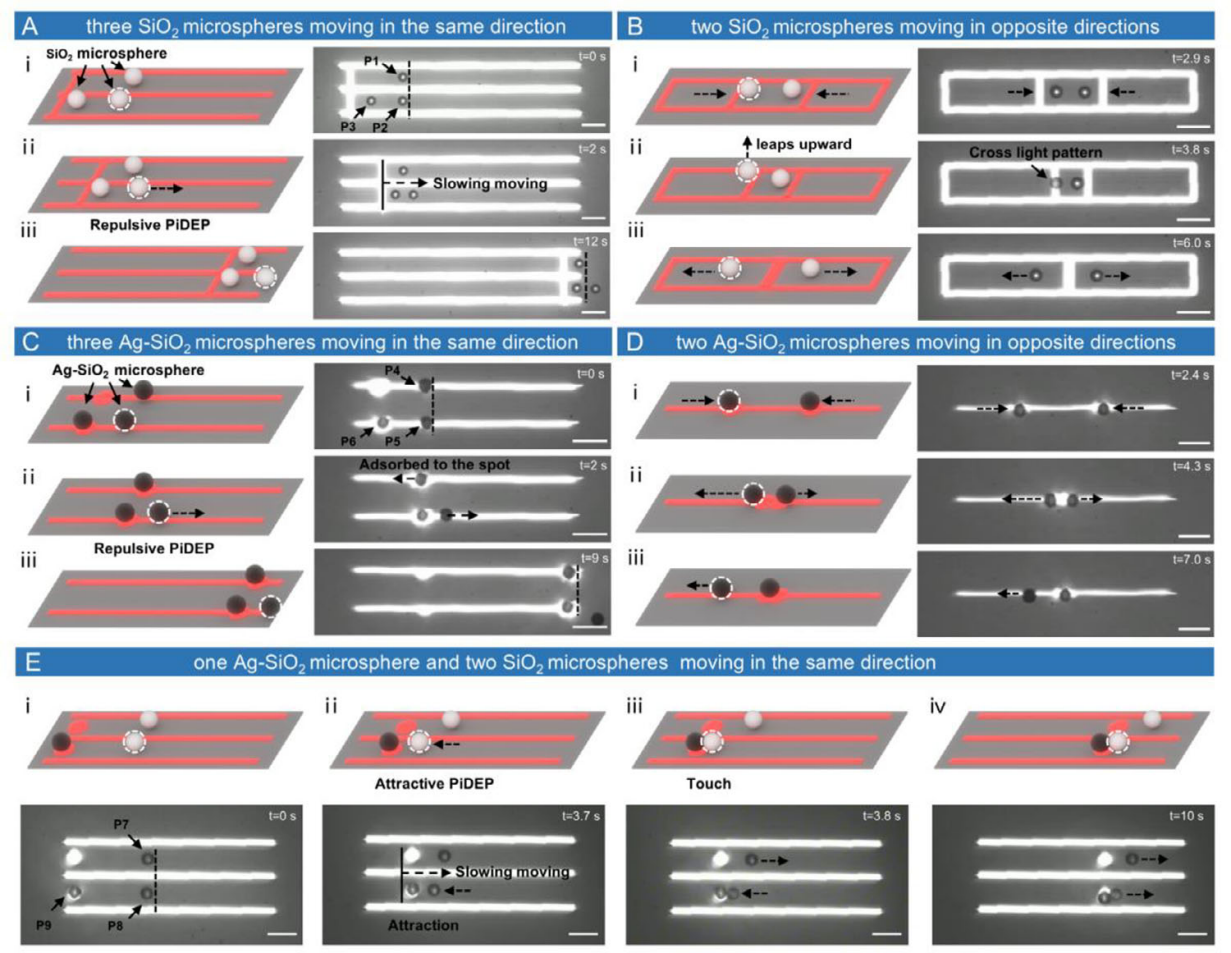
Particle interactions in the OET system. A) Comparison between OET‐driven and PiDEP‐driven SiO_2_ microspheres. (i) Microspheres P1 and P2 are placed on the same starting line. (ii) As the optical pattern moves slowly to the right, microsphere P2 begins to move to the right. (iii) Microsphere P2 moves ahead of microsphere P1. B) The OET drives two SiO_2_ microspheres toward each other. (i) The optical pattern pushes the SiO_2_ microspheres toward the center. (ii) The left microsphere leaps upward and passes over the optical pattern. (iii) The two microspheres move in opposite directions. C) Comparison between OET‐driven and PiDEP‐driven Ag‐SiO_2_ microspheres. (i) Microspheres P4 and P5 are aligned on the same starting line. (ii) Two light spots move slowly to the right; microsphere P4 is attracted into the closest light spot, while microsphere P5 moves to the right. (iii) Microsphere P4 moves ahead of microsphere P5. D) The OET drives two Ag‐SiO_2_ microspheres toward each other. (i) Light spots attract the Ag‐SiO_2_ microspheres toward the center. (ii) As the microspheres approach each other, PiDEP repulsion occurs. (iii) The Ag‐SiO_2_ microsphere on the left escapes the light spot and moves to the left, while the microsphere on the right remains trapped by the light spot. E) Interaction between SiO_2_ and Ag‐SiO_2_ microspheres. (i) Microspheres P7 and P8 are placed on the same starting line; the lower light spot carries an Ag‐SiO_2_ microsphere. (ii) Both light spots move slowly to the right; microsphere P8 moves to the left because of the attractive PiDEP, while microsphere P7 remains stationary. (iii) Microsphere P8 contacts P9, and microsphere P7 begins moving to the right. (iv) All microspheres move to the right simultaneously. All experiments used an AC signal of 50 kHz and 10 Vpp. The solution conductivity was 1.9 × 10^−3^ S m^−1^. Scale bar: 50 µm.

The experiment shown in **Figure** **3** was designed to investigate the PiDEP mechanism in the OET system. First, we used a light projection pattern to create two “tracks” (as shown in Figure 3A and Movie S2, Supporting Information). At *t*  =  0 s, SiO_2_ microspheres P1 and P2 were placed on the same starting line (indicated by the black dashed line in the figure). The strip‐shaped light pattern was then moved to the right at a constant speed of 20 µm s^−1^. We selected this low speed for the light pattern to avoid hydrodynamic interference during the “race.” As the SiO_2_ microspheres were subjected to a negative DEP force, the light pattern exerted a repulsive force on them, pushing them to the right. At *t*  =  2 s, P2 had already started moving to the right, whereas P1 remained stationary. This observation indicated that the force moving microsphere P2 was not the ODEP, but rather the repulsive PiDEP from microsphere P3 acting on P2. The effective range of this repulsive force was greater than that of the ODEP.

Next, we placed two SiO_2_ microspheres on the same track and used a light pattern to push them slowly toward the center (Figure 3B and Movie S3, Supporting Information). At *t* = 2.9 s, the speed of the microspheres decreased. By *t* = 3.8 s, the left microsphere escaped the effect of the ODEP and jumped upward through the light pattern, and then the right microsphere did the same. For SiO_2_ microspheres that experienced a negative DEP force, the ODEP had an upward component perpendicular to the chip, causing the particles to jump when passing through a light pattern.^[^ ^38^, ^60^ ^]^ Finally, at *t*  =  6.0 s, the two microspheres moved in opposite directions. This phenomenon suggested that as the distance between the microspheres decreased, the repulsive PiDEP increased, eventually surpassing the ODEP at a certain point.

We then studied the PiDEP interaction between particles subjected to a positive DEP force using the previously mentioned Ag‐SiO_2_ microspheres. We again created two “tracks” using light patterns (as shown in Figure 3C and Movie S4, Supporting Information). At *t* = 0 s, P4 and P5 were placed on the same starting line, two circular light spots were projected, and microsphere P6 was positioned at the lower spot. Both light spots were moved to the right at a constant speed of 20 µm s^−1^. At t = 2 s, P4 was attracted to the circular light spot, whereas P5 resisted this attraction and started moving to the right. This behavior indicated that P5 experienced a repulsive force from P6, proving that the PiDEP also occurred between particles subjected to a positive dielectrophoretic force.

Subsequently, we placed two Ag‐SiO_2_ microspheres on the same track, using two circular light spots to move them toward each other (as shown in Figure 3D and Movie S5, Supporting Information). At *t*  =  4.3 s, the two light spots converged into one, and the Ag‐SiO_2_ microspheres started moving in opposite directions owing to the repulsive force. By *t*  =  7.0 s, the left microsphere overcame the attraction of the light spot and rapidly moved toward the left. However, as the distance between the microspheres increased, the repulsive force quickly diminished, preventing the right microsphere from escaping the attraction of the light spot and trapping it. This observation indicated that for microspheres subjected to a positive DEP force, the repulsive PiDEP force increased as the distance between the microspheres decreased, and it eventually surpassed the ODEP force at a certain proximity. This behavior was the same as that observed for the SiO_2_ microspheres.

Figure 3E illustrates the interaction between the Ag‐SiO_2_ and SiO_2_ microspheres in the OET system (Movie S6, Supporting Information). At *t*  =  0 s, microspheres P7 and P8 were placed on the same starting line, and microsphere P9 was attracted to the light spot on the lower track. The light spots on both the upper and lower tracks moved to the right simultaneously at a speed of 20 µm s^−1^. At *t*  =  3.7 s, microsphere P8 moved to the left, while microsphere P7 remained stationary. By *t*  =  3.8 s, microsphere P8 was attracted to microsphere P9, whereas microsphere P7 was pushed to the right by the light spot. Subsequently, microsphere P9 carried microsphere P8 as they moved to the right together. This phenomenon indicated that in the horizontal direction, an electrostatic attraction existed between the microspheres subjected to a positive DEP force and those subjected to a negative DEP force.

**Figure 4:**
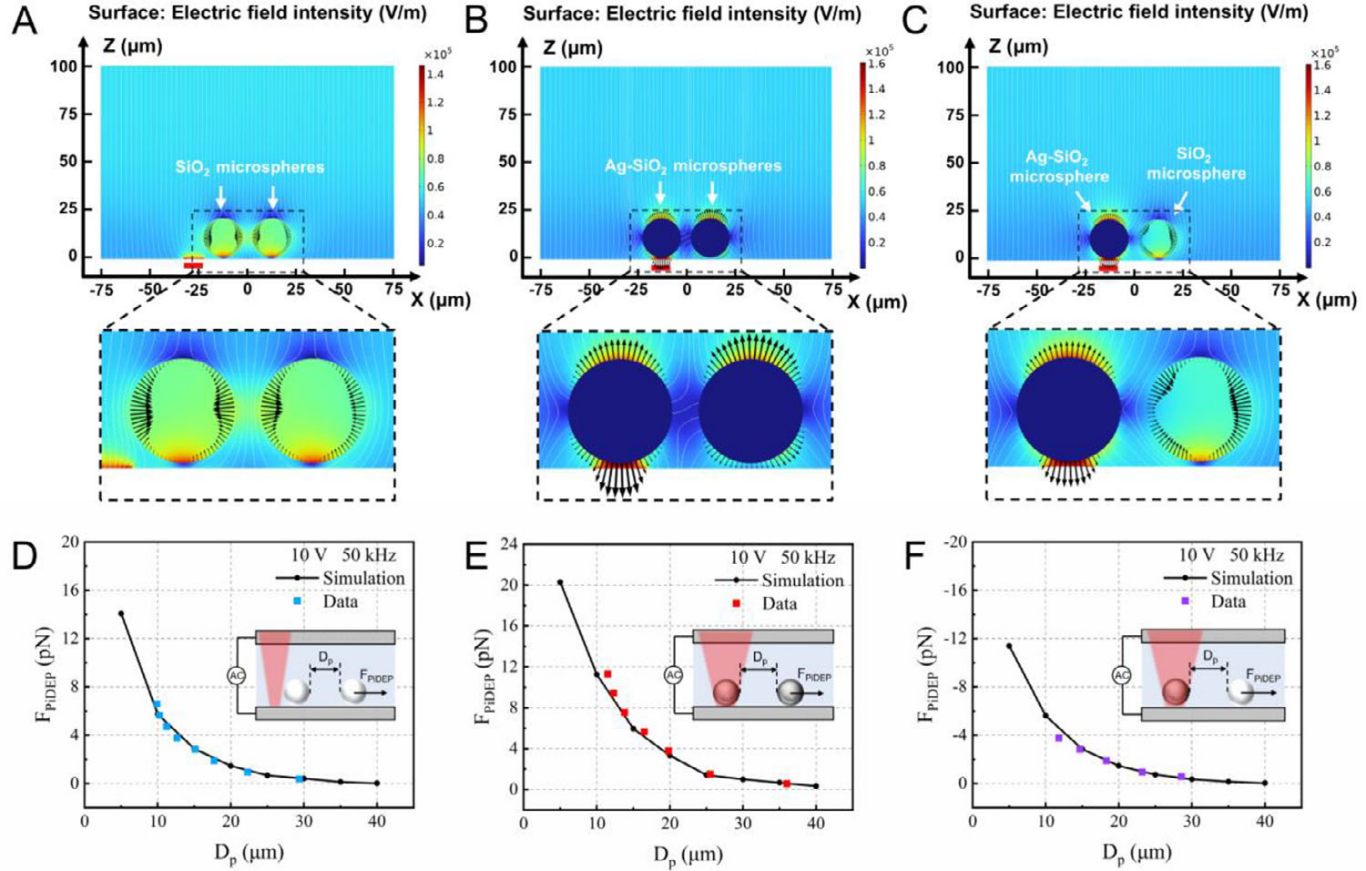
Simulation analysis of the PiDEP between particles. A–C) AC electric field strength (blue indicates low, red indicates high) for two SiO_2_ microspheres, two Ag‐SiO_2_ microspheres, and one Ag‐SiO_2_ microsphere and SiO_2_ one microsphere, respectively. Black arrows represent the Maxwell stress tensor on the surface of the microspheres, and white streamlines show the direction of the electric field. The red mark at the bottom denotes the width of the irradiation area of the light spot. D–F) Variation in the PiDEP as a function of the microsphere distance for (A)–(C), respectively. The solid lines represent the simulation results, and the squares represent the experimental data.

To more effectively explain the experimentally observed phenomena, we developed a numerical simulation model (Figure S4, Supporting Information) and simulated the three particle interaction scenarios presented in Figure 3. The simulation results (**Figure** **4**) revealed that electric field polarization redistributed surface charges on the particles, thereby altering the local electric field. When two particles were in close proximity, an uneven electric field was generated between the particles, resulting in PiDEP acting between them.

Since the particles affected the surrounding electric field, they could not be approximated as electric dipoles. Therefore, we used the Maxwell stress tensor method^[^ ^28^, ^54^, ^61^ ^]^ to accurately calculate the PiDEP (Note S4, Supporting Information). The PiDEP between the two Ag‐SiO_2_ microspheres or two SiO_2_ microspheres was positive, indicating repulsion. The PiDEP between the Ag‐SiO_2_ and SiO_2_ microspheres was negative, indicating attraction. This result was consistent with the experimental observations shown in Figure 3. In addition, by measuring the speed of the microspheres in the experiment, the PiDEP acting on the microspheres was calculated using the Stokes drag formula (Note S5, Supporting Information). The results showed that the numerical simulation was consistent with the experimental data (Figure 4D–F). Moreover, the magnitude and effective range of the PiDEP between the Ag‐SiO_2_ microspheres were the largest among the three cases, which may be attributed to the high dielectric constant of metallic silver, leading to an expanded region of electric field influence.

It is well known that even in the absence of an external electric field, surface charges can exist on microparticles. The type and density of these charges can be characterized by the zeta potential. Both Ag‐SiO_2_ and SiO_2_ microspheres exhibited negative zeta potentials of −27.8 and −30.2 mV, respectively. After amination surface modification, the NH_2_@Ag‐SiO_2_ and NH_2_@SiO_2_ microspheres were obtained, with zeta potentials of +15.3 and +20.4 mV, respectively (Figure S5A and Note S6, Supporting Information). Comparative experiments showed that whether the zeta potential was positive or negative had no noticeable effect on the strength of PiDEP or the performance of particle‐assisted OET (Figure S5B, Supporting Information).

In summary, the particles subjected to the same type of DEP force experienced PiDEP repulsion, whereas those subjected to opposite types of DEP forces experienced PiDEP attraction. This behavior was not affected by the zeta potential of the microspheres. Moreover, PiDEP can coexist with OET and exerts a stronger influence. Notably, PiDEP can propagate among multiple microparticles (Figure S6A and Movie S7, Supporting Information). By leveraging particle‐assisted OET, microparticles can be indirectly controlled, enabling them to navigate around obstacles (Figure S6B and Movie S8, Supporting Information).

### 2.3 Indirect Cell Manipulation Using Particle‐Assisted OET

**Figure 5:**
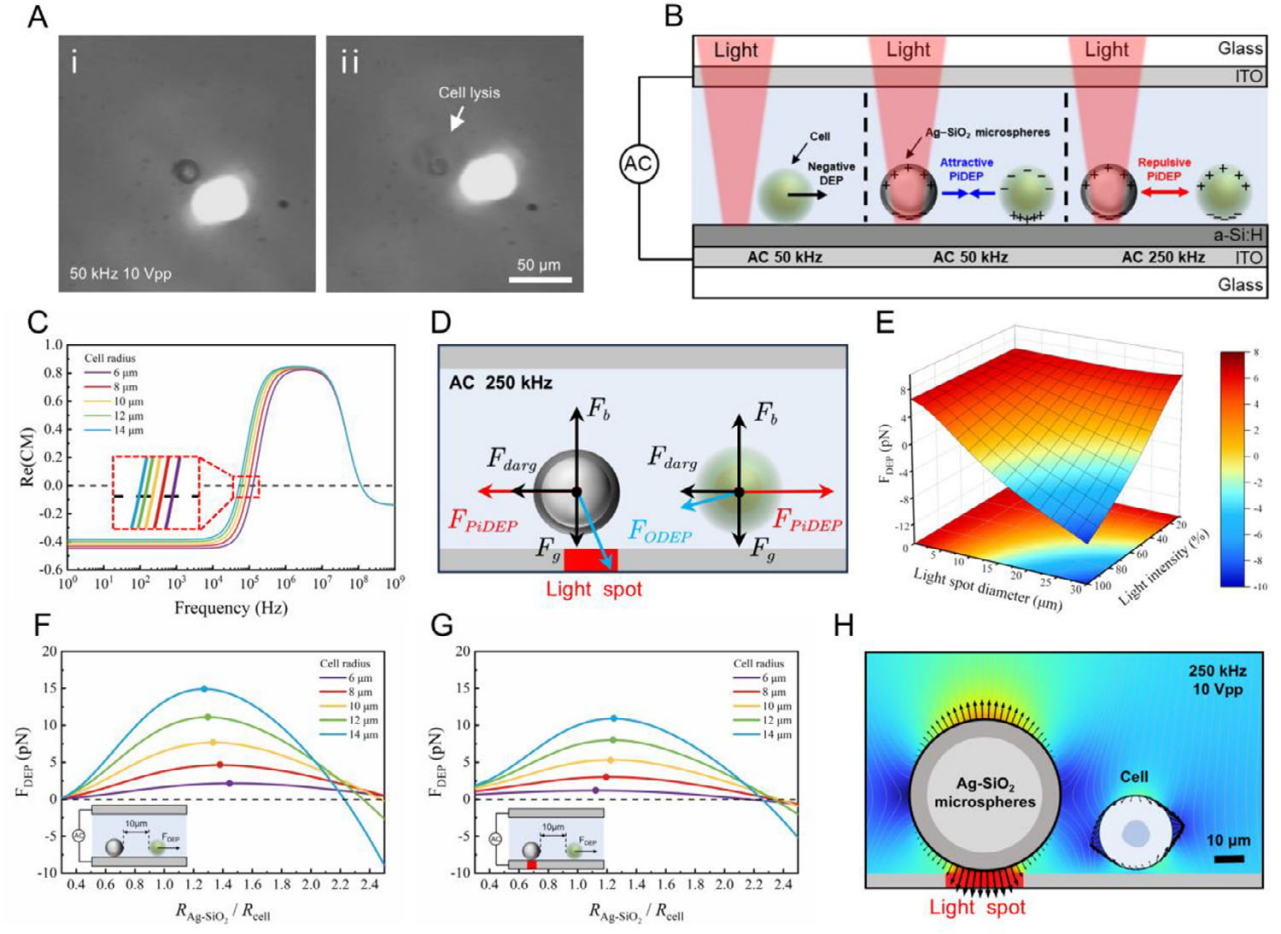
Simulation of factors influencing particle‐assisted OET for cell manipulation. A) Cell lysis and death of 293T cells induced by conventional OET manipulation. B) Schematic of particle‐assisted OET for cell manipulation and operational strategy. C) Real part of the Clausius–Mossotti factor for cells with different radii. D) Force analysis diagram of a cell during particle‐assisted OET manipulation. E) Effect of light spot diameter and light intensity on the total DEP force acting on a cell (cell and microsphere radius: 10 µm; surface‐to‐surface distance: 10 µm; positive values indicate repulsion, negative values indicate attraction). F,G) Total DEP force on the cell as a function of Ag‐SiO_2_ particle radius under illuminated and non‐illuminated conditions, respectively. (The solid dots indicate the maximum values of the curves.) H) Electric field intensity and Maxwell stress tensor distribution for particle‐to‐cell radius ratios of 2.

When using OET for direct cell manipulation, the light spot must be very close to the cell to generate a sufficiently large DEP force that can move the cell. This is because the gradient of the electric field modulus is highest at the boundary between the illuminated and dark regions of the chip, where the DEP force peaks.^[^ ^62^ ^]^ However, the electric field in the illuminated area can significantly increase the transmembrane potential of the cell,^[^ ^63^, ^64^ ^]^ possibly causing cell lysis and death (**Figure** **5**A and Movie S9, Supporting Information), particularly at lower AC frequencies.^[^ ^65^, ^66^ ^]^

To address this challenge, we developed an indirect cell manipulation method using particle‐assisted OET, and demonstrated it using 293T cells. First, we adjusted the electrical parameters so that both the 293T cells and Ag‐SiO_2_ microspheres experienced the same type of DEP force, making the PiDEP repulsive between them (Figure 5B). Using the method described in the literature for measuring the crossover frequency of cells,^[^ ^23^ ^]^ the crossover frequency of 293T cells was determined to be ≈100 kHz in a solution with a conductivity of 2.5 × 10^−2^ S m^−1^. We used a shell–core equivalent model to calculate the Re(*K*) values of cells with different radii, and the theoretical crossover frequencies obtained were consistent with the experimental results (Figure 5C). Therefore, 293T cells transitioned from experiencing to negative DEP force to positive DEP force by adjusting the AC frequency from 50 to 250 kHz (Movie S10, Supporting Information).

The motion of cells in a fluid medium involves complex dynamic behaviors. This study simplifies the analysis by neglecting cell rotation and friction with the OET chip surface, focusing on a quasi‐static force analysis (Figure 5D). During particle‐assisted OET manipulation, the cell is primarily subjected to DEP force, hydrodynamic drag, gravity, and buoyancy forces. The total DEP force comprises ODEP and PiDEP, which are nonlinearly superimposed.

To identify optimal manipulation parameters for particle‐assisted OET, a multiphysics simulation model was developed. First, the influence of optical parameters (spot diameter and intensity) were investigated. As shown in Figure 5E, increasing light spot diameter and intensity enhances ODEP dominance over PiDEP, shifting the total DEP force from repulsion to attraction. Thus, we selected moderate optical settings (10 µm light spot diameter and 50% light intensity) to achieve the best balance for stable and effective control. In addition, the size ratio between the cell and the metal microsphere significantly impacts the manipulation performance. Figure 5F,G demonstrates that reducing cell size consistently weakens the total DEP force, regardless of illumination conditions. Meanwhile, as the radius ratio between the microsphere and the cell $\left(R_{Ag-SiO_{2}}/R_{cell}\right)$ increases, the total DEP force initially increases and then decreases, peaking at a ratio of 1.0–1.3. Notably, when the microsphere radius exceeds twice the cell radius, the cell may be attracted to the bottom of the microsphere due to a mismatch in the polarization direction along the horizontal axis (Figure 5H). This phenomenon should be avoided in practical applications to ensure precise cell positioning. Considering that 293T cells have diameters between 15 and 20 µm, 20 µm Ag‐SiO_2_ microspheres were a relatively suitable option for particle‐assisted OET manipulation.

**Figure 6:**
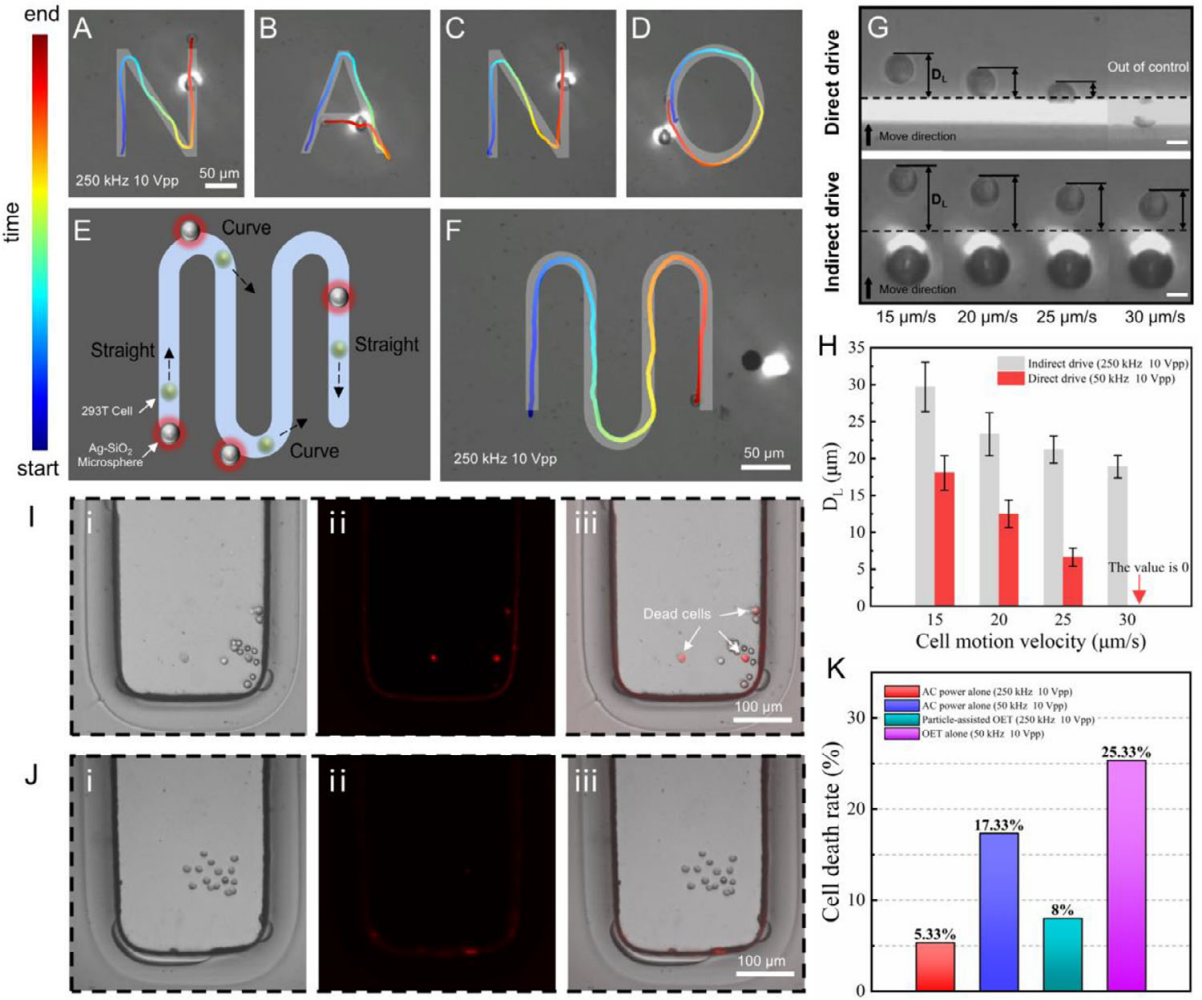
Single‐cell manipulation using particle‐assisted OET. A–D) Time‐lapse images showing a 293T cell moving along a “NANO” trajectory driven by particle‐assisted OET. E,F) Schematic and time‐lapse micrographs, respectively, of a 293T cell navigating straight and curved paths driven by particle‐assisted OET. The starting point is marked in blue and the ending point is marked in red. G,H) Comparison of the distance between the light spot and 293T cells when using particle‐assisted OET versus OET alone for different speeds. Scale bar: 10 µm. The bright‐field, fluorescence, and I,J) overlay images of 293T cells manipulated by OET alone and particle‐assisted OET. K) Mortality rate of 293T cells for different experimental parameters. The cell death rate was assessed after 10 min on the OET chip under the electrical conditions of 10 Vpp at 250 kHz (red hatch, *n* = 4/75) and 50 kHz (blue hatch, *n* = 13/75). The cell death rate was evaluated after 120 s of manipulation using particle‐assisted OET (turquoise hatch, *n* = 6/75) and OET alone (violet hatch, *n* = 19/75). The numbers of cells evaluated in each condition (*n*) were pooled from five replicates of 15 cells.

**Figure** **6**A–D and Movie S11 (Supporting Information) show the 293T cell trajectory that was manipulated using this indirect method, forming the word “NANO.” The indirect manipulation enabled the cells to perform sharp turns, sudden stops, and curved movements. Figure 6E,F shows an “S”‐shaped trajectory formed by the indirect manipulation, with the cells smoothly shifting between straight and curved paths (Movie S12, Supporting Information). As shown in Figure 6G,H, the particle‐assisted OET increased the manipulation distance by 2–3 times compared to the conventional OET. Moreover, at a speed of 30 µm s^−1^, the conventional OET was unable to control the cell, but the cell manipulation of the particle‐assisted OET remained effective. Notably, in addition to Ag‐SiO_2_ microspheres, microspheres coated with other metals such as gold and nickel also exhibited comparable manipulation performance (Note S7, Supporting Information).

We conducted a cell viability experiment to assess the effects of the particle‐assisted OET on the cells (Figure 6I,J, and Note S8, Supporting Information). As shown in Figure 6K, the cell death rate that occurred when using the particle‐assisted OET was reduced by a factor of three compared to that when using the OET alone, decreasing from 25.33% to 8%. This improvement was attributed to two factors. First, the light spot was farther from the cell, reducing the damage caused by its light and heat. Second, the particle‐assisted OET operated at a higher AC frequency, which decreased the harmful effects of the electric field on the cell.

In all the experimental cases, the light spot was manually controlled. Although manual operation offered higher flexibility, its precision was inferior to that of the direct OET manipulation. The particle‐assisted OET was an inherently unstable control system, similar to an inverted pendulum. However, by integrating automatic recognition with advanced control algorithms, precise trajectory control can be achieved. We have made preliminary progress in parallel manipulation of a small number of microparticles by particle‐assisted OET,^[^ ^67^ ^]^ but large‐scale parallel control of cells remains a key focus of our future research.

## 3 Discussion

In this study, we proposed a method for indirectly manipulating single cells and microparticles using particle‐assisted OET, effectively addressing the issue of cell damage caused by traditional OET. By manipulating conductive microspheres, the effective manipulation range of the OET for target objects was increased two to three times. This approach avoided directly illuminating the cells with the manipulating light and reduced the damage caused to them by the electric field, thereby improving the cell viability. Compared to other optical tweezer–based indirect manipulation strategies,^[^ ^45^, ^46^, ^47^ ^]^ particle‐assisted OET enables gentle and effective cell manipulation under significantly lower optical power densities (≈0.4 mW cm^−2^), which are several orders of magnitude lower than those typically required for conventional optical tweezers (≈10^6^ mW cm^−2^). Despite these advantages, the large‐scale application of this technology in the biological field still faces several challenges, including the need for advanced control algorithms to ensure precise and reliable manipulation of cells and particles, as well as the adaptability of the technology to a wide variety of organisms and cell types. We will continue to conduct research on these challenges.

In addition, this technology could be applied not only to the manipulation of living cells but also to the light‐sensitive objects, such as fluorescent microspheres and photosensitive gel microspheres. Furthermore, this PiDEP‐based manipulation strategy can be integrated with optical tweezers, magnetic control technologies, and other methods to further expand micro‐ and nanomanipulation techniques.

## 4 Experimental Section

### Experimental Setup

The OET system (Light Operator S1, Micro Nano Robot Co., Ltd.), shown in Figure 1C, consisted of an optical setup, chip holder, XYZ motion platform, and injection pump (RUNZE, SY03B), which followed a previously reported experimental design.^[^ ^68^ ^]^ The chip holder was connected to the injection pump via a capillary tube, and a silicone stopper was used to seal the OET chip, eliminating the conventional need for polydimethylsiloxane (PDMS) stoppers.^[^ ^25^ ^]^ In addition, the chip holder featured a Peltier heater that maintained the OET chip at a constant temperature of 37 °C. A signal generator (UTG2062B) was connected to the chip holder via a coaxial cable, and an AC signal was supplied to the OET chip. The XYZ motion platform held the OET chip and enabled precise positioning and automatic focusing. The optical setup adopted an upper illumination structure that included projection, illumination, and observation optical paths (Figure 1D). The projection pattern, generated by a digital micromirror device (DMD), was projected through the projection lens and a semi‐reflective mirror into a microscope objective (Olympus MPLFLN10X). The illumination was generated using an LED point source. After passing through two convex lenses, it became approximately collimated and was then coupled to the projection optical path via a semireflective mirror. The projection light wavelength was 610 nm and the illumination wavelength was 580 nm. An optical power meter was used to measure the projection light power (Sorebo PM121D), which reached a maximum optical power density of 0.8 W cm^−2^ under the 10× objective lens and a projection accuracy of 0.4 µm for single linewidths. The OET chip was encapsulated on a printed circuit board (PCB), allowing different channel shapes to be replaced, as required by the experiments. The conductivity of the solutions was measured using a conductivity meter (DDSJ/318T, Shanghai Lei Magnetic).

### Sample Preparation

SiO_2_ microspheres (20 µm in diameter) were supplied as an aqueous suspension (MicroNano Intelligent Technology Co., Ltd.). Ag‐SiO_2_ spheres were prepared via electroless plating, forming a silver shell thickness of 80–100 nm.^[^ ^69^ ^]^ The microspheres were resuspended in 0.1% (v/v) Tween‐20 (Sigma Aldrich). 293T cells were cultured in Dulbecco's Modified Eagle Medium (DMEM, Feimobio Life Sciences, China) containing 10% (v/v) fetal bovine serum (FBS, Feimobio Life Sciences, China) and 1% (v/v) penicillin‐streptomycin, and maintained in a cell culture incubator at a temperature of 37 °C and a CO_2_ level of 5%.^[^ ^38^ ^]^ Before each experiment, the cells were washed twice in phosphate‐buffered saline (PBS) solution (Feimobio Life Sciences, China) and resuspended in sucrose buffer (DI water, 8.5% (w/v) sucrose, and 0.3% (w/v) glucose (conductivity: 2–3 × 10^−2^ S m^−1^) at 5 × 10^5^ cells mL^−1^. Each suspension was filtered using a 40‐µm cell strainer (Corning).

### Fabrication of the OET Chip

The OET chip primarily consisted of two ITO‐coated glass slides and a microchannel layer. The upper and lower glass layers were 0.3 mm and 0.7 mm thick, respectively, and each was coated with a 200‐nm layer of ITO (HDXCKJ Supplies). The microchannel layer was fabricated using a two‐step in situ photolithography process, as illustrated in Figure S8 (Supporting Information). Briefly, a bottom microchannel structure was prepared (Figure S8A, Supporting Information). A layer of a‐Si:H with a thickness of 1 µm was deposited on the lower layer of the ITO‐coated glass using plasma‐enhanced chemical vapor deposition (PECVD), followed by a 20‐nm silicon nitride layer for passivation. A photoresist SU‐8 2025 (Microchem) was spin‐coated at 2000 rpm and soft‐baked at 95 °C for 5 min. The mask was aligned to the lower glass substrate using a photolithography machine and exposed (12 mJ cm^−2^ for 7 s). Post‐exposure, the substrate underwent a post‐bake at 95 °C for 8 min and was then developed in SU‐8 developer (Microchem) for 5 min. The developed substrate was rinsed with isopropanol and deionized water, and then dried with nitrogen gas. Finally, the films were hardened at 200 °C for 5 min. The upper bonding layer shown in Figure S8B (Supporting Information) was then prepared. Laser cutting was used to create through‐holes with a diameter of 50 µm in the upper ITO glass, serving as inlet and outlet ports. The processed glass was ultrasonically cleaned with anhydrous ethanol and deionized water to remove debris, and then dried in a hot oven. A layer of NOA61 adhesive (≈5 µm thick) was spin‐coated onto the substrate at 2000 rpm and prebaked at 100 °C for 5 min. As illustrated in Figure S8C (Supporting Information), the NOA61‐coated ITO glass was aligned with and pressed onto the SU‐8 microchannel. The same mask from Figure S8A (Supporting Information) was used for in situ secondary photolithography (exposure energy: 12 mJ cm^−2^, time: 10 s). After developing in anhydrous ethanol for 3 min, an injection pump flushed the microchannel with anhydrous ethanol through the inlet to remove any residual NOA61. This two‐step in situ photolithography and bonding process not only overcame the weak bonding capability of the SU‐8 photoresist, but also allowed the NOA61 to fill the gaps between the SU‐8 microchannel and the upper ITO layer, improving the sealing of the chip's microchannel.

### Image Processing and Particle Tracking

All images were captured using a charge‐coupled device (CCD) camera (Alvium, U‐1240 m). Movie data were processed using ImageJ and Python (version 3.9.7) to extract the trajectories of the microspheres and cells. The particle velocities were calculated based on the scale of the system and the time intervals between the image frames.

## Conflict of Interest

The authors declare no conflict of interest.

## Supporting information



Supporting Information

Supplemental Movie 1

Supplemental Movie 2

Supplemental Movie 3

Supplemental Movie 4

Supplemental Movie 5

Supplemental Movie 6

Supplemental Movie 7

Supplemental Movie 8

Supplemental Movie 9

Supplemental Movie 10

Supplemental Movie 11

Supplemental Movie 12

## Data Availability

The data that support the findings of this study are available from the corresponding author upon reasonable request.
