# Supplementary material for: Particle‐Assisted Optoelectronic Tweezers for Manipulating Single Cells and Microparticles
Source: Adv Sci (Weinh). 2025 May 5;12(26):2501032. doi: 10.1002/advs.202501032 (PMC12245005; doi:10.1002/advs.202501032)
Supplement: Supplementary file 1 — Supporting Information [file ADVS-12-2501032-s013.docx]

Supporting Information

Particle-assisted Optoelectronic Tweezers for Manipulating Single Cells and Microparticles

Ao Wang, Shuzhang Liang, Caiding Ni, Yongyi Jia, Kangjie Wu, Wenyan Niu, Shunxiao Huang, Kaiyi Peng, Chutian Wang, Yingjian Guo, Zhijun Zhao, Lingze Zhang*, Mingjie Liu*, and Lin Feng*

**The PDF file includes:**

Supplementary Notes S1 to S8

Figures. S1 to S8

Tables S1 to S3

References

**Other Supplementary Materials for this manuscript include the following:**

**Movie S1.**

The light spot approaches the SiO2 and Ag-SiO2 microspheres from left to right under OET control.

**Movie S2.**

Comparison of OET-driven and PiDEP-driven SiO2 microspheres.

**Movie S3.**

The OET drives the two SiO2 microspheres toward each other.

**Movie S4.**

Comparison of OET-driven and PiDEP-driven Ag-SiO2 microspheres.

**Movie S5.**

The OET drives the two Ag-SiO2 microspheres toward each other.

**Movie S6.**

Interaction between SiO2 and Ag-SiO2 microspheres.

**Movie S7.**

The particle-assisted OET drives multiple SiO2 microspheres along a light path with a 90° corner.

**Movie S8.**

Transportation of an Ag-SiO2 microsphere around obstacles using the particle-assisted OET.

**Movie S9.**

293T cell lysis during direct manipulation by the OET.

**Movie S10.**

293T cell transition from experiencing negative DEP force to positive DEP force by changing the AC frequency.

**Movie S11.**

293T cell moving along a "NANO" trajectory driven by the particle-assisted OET.

**Movie S12.**

293T cell navigating straight and curved paths driven by the particle-assisted OET.

**Text:**

**Supplementary Note S1:** Calculation of Re(*K*)

For spherical particles composed of a single material, such as SiO_2_ microspheres, Re(*K*) can be calculated using

$K=\frac{\varepsilon_{p}^{*}-\varepsilon_{m}^{*}}{\varepsilon_{p}^{*}+2\varepsilon_{m}^{*}}$ (S1)

where $\varepsilon_{p}^{\text{*}}$ and $\varepsilon_{m}^{*}$ are the complex permittivities of the particle and medium, respectively, which are defined as $\varepsilon^{\text{*}}=\varepsilon-i\sigma/\omega$, where $\varepsilon$ is the permittivity, $\sigma$ is the electrical conductivity, $\omega=2\pi f$ is the angular frequency, and $i$ is the imaginary unit.

For shell-type particles, such as Ag-SiO_2_ microspheres or cells, Re(*K*) can be calculated using

$K=\frac{\varepsilon_{core-shell}^{\text{*}}-\varepsilon_{m}^{\text{*}}}{\varepsilon_{core-shell}^{\text{*}}+2\varepsilon_{m}^{\text{*}}}$ (S2)

$\varepsilon_{core-shell }^{*}=\varepsilon_{shell}^{*}\cdot\frac{\left(\frac{R}{r}\right)^{3}+2\left(\frac{\varepsilon_{core}^{*}-\varepsilon_{shell}^{*}}{\varepsilon_{core}^{*}+2\varepsilon_{shell}^{*}}\right)}{\left(\frac{R}{r}\right)^{3}-\left(\frac{\varepsilon_{core}^{*}-\varepsilon_{shell}^{*}}{\varepsilon_{core}^{*}+2\varepsilon_{shell}^{*}}\right)}$ (S3)

where $R$ and $r$ are the outer and inner radii of the Ag-SiO_2_ microspheres, respectively, and $\varepsilon_{shell}^{\text{*}}$ and $\varepsilon_{core}^{\text{*}}$ are the complex permittivities of the shell and core, respectively.

Using the parameters listed in Table S1 and S2, we calculated Re(*K*) for both the Ag-SiO_2_ and SiO_2_ microspheres (Figure 2D, the main text), as well as for different radius of cells (Figure 5C, the main text).

**Supplementary Note S2:** Measurement of photoconductivity and dark conductivity of the a-Si:H film

We measured the photoconductivity and dark conductivity of the photoconductive layer in the OET chip. First, an array of 1 mm × 1 mm square gold electrodes was deposited onto the surface of the a-Si:H film using magnetron sputtering, with a spacing of 1 mm between adjacent electrodes. Next, the a-Si:H film between neighboring electrodes was characterized using a semiconductor parameter analyzer. The resulting photocurrent and dark current curves are shown in Figure S1. Under an applied voltage of 10 V, the photocurrent reached 3.3 × 10^-9^ A, while the dark current was 6.7 × 10^-12^ A.

The conductivity of the a-Si:H film was calculated using the following equation:

$\sigma=\frac{I\cdot L}{V\cdot A_{c}}$ (S4)

where *L* is the electrode spacing (1 mm), and *A_c_* is the cross-sectional area (1 µm × 1 mm).

The calculated photoconductivity and dark conductivity of the photoconductive layer in the OET chip are 3.3 × 10^-4^ S/m and 6.7 × 10^-7^ S/m, respectively.

**Supplementary Note S3:** Numerical Simulation of Circular Light Spots in the OET

The 3D simulation was performed using the AC/DC module of COMSOL Multiphysics (version 5.5). As illustrated in Figure S2, the model dimensions were 100 μm long (X-axis), 100 μm wide (Y-axis), and 100 μm high (Z-axis). The model consisted of a 99-μm-thick liquid chamber above a 1-μm-thick a-Si:H film at the bottom. At the center of the model, a circular area with a diameter of 30 μm simulated the illuminated region of the light pattern (depicted in red), and its conductivity was assigned to be two orders of magnitude higher than that of the a-Si:H film in darkness.

In this model, the boundary conditions were defined using electrical insulation for all external edges and electrical continuity for all internal boundaries. The top was grounded and the bottom was set to 10 V to simulate an AC signal at a frequency of 50 kHz. The conductivities and dielectric constants of the solution medium and a-Si:H used in the model are listed in Table S3. As noted in Supplementary Note S2, the photoconductivity and dark conductivity of the a-Si:H film were measured. To simplify the simulation process, the measured values were approximated by rounding.

**Supplementary Note S4:** Numerical simulation of PiDEP between two particles in the OET

Three-dimensional simulations were conducted using the AC/DC module of COMSOL Multiphysics (version 5.5). As shown in Figure S4, the simulation model dimensions were 200 μm long (X-axis), 100 μm wide (Y-axis), and 100 μm high (Z-axis). The model included a 99-μm-thick liquid chamber above a 1-μm-thick a-Si:H film at the bottom. The model contained two microspheres with a diameter of 20 μm to simulate the SiO_2_ and Ag-SiO_2_ microspheres (the Ag-SiO_2_ microspheres had an outer silver shell 100 nm thick). The two microspheres were positioned near the bottom of the liquid chamber, with their centers located in the XZ plane 100 nm above the surface of the a-Si layer. To simulate the illuminated area of the light pattern, we introduced a circular region with a diameter of 10 μm directly beneath microsphere 1 (depicted as the red region in the model). This region represents the area in which light increased the conductivity of the a-Si layer, simulating the effect of illumination in the OET system.

In this model, the boundary conditions were defined using electrical insulation on all external edges and electrical continuity on all internal boundaries. The top was grounded and an AC signal of 10 V at 50 kHz was applied to the bottom. Depending on the simulation requirements, the materials of the two microspheres were set to either SiO_2_ or Ag; their conductivities and dielectric constants are listed in Table S1. The conductivity and dielectric constant settings for the solution medium and a-Si:H used in the model are listed in Table S2. The electric field intensity of the cut plane shown in Figure S4 (XZ slice at Y=0) was simulated, as shown in Figures 5A-C in the main text.

The Maxwell stress tensor was used to calculate the DEP force acting on the particles to account for their influence on the surrounding electric field. The Maxwell stress tensor is defined as

$\sigma^{\text{MST}}=\varepsilon^{\text{*}}\left(EE-\frac{1}{2}\left|E\right|^{2}I\right)+\mu^{\text{*}}\left(HH-\frac{1}{2}\left|H\right|^{2}I\right)$ (S5)

where $\varepsilon^{\text{*}}$ represents the complex permittivity, $\mu^{\text{*}}$ represents the complex permeability, $E$ represents the electric field intensity, $H$ represents the magnetic field intensity, and $I$ represents the unit tensor.

The volumetric force on a dielectric object is expressed as

$F=\text{∮}_{S} \sigma^{\text{MST}}\cdot ndS$ (S6)

where *S* represents the surface of the particle and ***n*** is the unit vector normal to the surface.

Neglecting the influence of the magnetic field ($H=0$), the stress tensor can be simplified to

$\sigma^{\text{MST}}=\varepsilon^{\text{*}}EE-\frac{1}{2}\varepsilon^{\text{*}}\left|E\right|^{2}I$ (S7)

The time-averaged stress tensor $\langle\sigma^{\text{MST}}\rangle$ for an alternating electric field is expressed as

$\langle\sigma^{\text{MST}}\rangle=\frac{1}{4}\text{Re}[\varepsilon^{*}]\left(EE^{'}+E^{'}E-|E|^{2}I\right)$ (S8)

where is the complex conjugate of the electric field .

To evaluate the DEP force, the integration surface was defined as the interface between the particle and the surrounding liquid medium, and the electric field used in the Maxwell stress tensor calculations was based on the properties of the fluid medium:

$\langle\sigma^{\text{MST}}\rangle=\frac{1}{4}\text{Re}\left[\varepsilon_{m}^{\text{*}}\right]\left(EE'+E'E-|E|^{2}\mathbf{I}\right)=\frac{1}{4}\varepsilon_{\text{m}}\left(EE'+E'E-|E|^{2}\mathbf{I}\right)$ (S9)

$F_{\text{dep}}=\text{∮}_{S} \langle\sigma^{\text{MST}}\rangle\cdot ndS$ (S10)

To calculate the DEP force on the microspheres in our simulations, the distance between the two microspheres was varied from 5 μm to 40 μm and sampled at 5 μm intervals. The simulated PiDEP was stronger than the force measured in the actual system. This discrepancy suggested that during the experiment, the microspheres may have experienced frictional forces or undergone other interactions with the surface^[1-2]^. After scaling the simulation data by factors of 3.2, 1.2, and 1.9 for the respective cases, the adjusted simulation results (shown in Figures 4D–F) exhibited qualitative agreement with the measured results.

**Supplementary Note S5:** OET system's performance in manipulating the SiO_2_ and Ag-SiO_2_ microspheres

In this study, the fluid system was in the laminar flow state (Reynolds number Re << 1). Under uniform motion conditions, the DEP force acting on the microspheres or cells was approximately equal to the viscous drag force^[3]^ and was calculated using Stokes' law, which is expressed as

$F_{DEP}=F_{drag}=6\pi\eta rv$ (S11)

where $\eta$ represents the fluid viscosity, $r$ is the radius of the microsphere or cell, and $v$ is the velocity of the microsphere or cell. Because the fluid was water and the experiments were conducted at room temperature, $\eta$ was assumed to be 1.0×10^-3^ (*Pa·S*).

**Supplementary Note S6:** The influence of Zeta potential on PiDEP

We modified the Ag-SiO_2_ and SiO_2_ microspheres surface with amino groups to obtain NH₂@Ag-SiO_2_ and NH₂@SiO_2_ microspheres. As shown in Figure S5A, the zeta potentials of Ag-SiO_2_, SiO_2_, NH_2_@Ag-SiO_2_, and NH_2_@SiO_2_ microspheres were measured in water (conductivity: 3 × 10^-3^ S/m, pH 7, temperature: 25 °C). Ag-SiO_2_ and SiO_2_ microspheres showed negative zeta potentials, while the modified NH₂@Ag-SiO_2_ and NH₂@SiO_2_ microspheres had positive values, indicating that the surface charges changed after modification. Subsequently, we used particle-assisted OET to manipulate Ag-SiO_2_ microspheres to drive the NH_2_@Ag-SiO_2_ microspheres, and SiO_2_ microspheres to drive NH_2_@SiO_2_ microspheres. As shown in Figure S5B, the modified microspheres experienced repulsive PiDEP forces similar to those observed with unmodified ones, and their manipulation speeds and repulsion forces were also comparable. These results suggest that the sign of the zeta potential does not affect the PiDEP strength or the effectiveness of particle-assisted OET.

**Supplementary Note S7:** Manipulation performance of microspheres coated with different metals

The results (Figure S7) show that polystyrene microspheres coated with gold (Au-PS) or nickel (Ni-PS) exhibit comparable maximum manipulation speeds to Ag-SiO_2_ microspheres.

This similarity can be attributed to the electrical conductivities of silver (σ = 6.3 × 10^7^ S/m), gold (σ = 4.1 × 10^7^ S/m), and nickel (σ = 1.4 × 10^7^ S/m) far exceed that of the solution medium (σ_sol_ = 3 × 10^-2^ S/m). Moreover, the metal shell acts as an effective shield, preventing the core material from influencing the particle's polarization behavior. As a result, their Re(*K*) values are all equal to 1, indicating that they exert the same level of polarization on nearby particles.

While Ag-SiO_2_ microspheres were used as a representative example, our results confirm that any metal coating with σ > 10^6^ S/m (e.g., Au, Ni, Cu) can serve as an effective intermediary. Moreover, the choice of core material does not affect the performance of particle-assisted OET. This flexibility allows researchers to select materials based on cost, biocompatibility, or commercial availability.

**Supplementary Note S8:** Comparison between viabilities of 293T cells manipulated by particle-assisted OET and OET alone

The buffer for 293T cells was prepared as described in the main text, except that cell staining reagents were not added. To detect dead cells, 5 µL of propidium iodide (PI) solution (Beyotime) was added to the 200 µL of the 293T cell suspension. Cell viability experiments were conducted under two different sets of conditions. First, to determine the number of dead cells, the cells were treated with an AC signal on the OET chip for 10 min at 250 kHz and 10 Vpp and at 50 kHz and 10 Vpp, followed by fluorescence microscopy. Second, the cells from the first step were manipulated using particle-assisted OET and OET alone for 120 s. Following this manipulation, fluorescence observations were conducted to record the number of dead cells.





**Figure S1.** Photocurrent and dark current of a-Si:H thin film. The light source used for illumination had a wavelength of 610 nm and an optical power density of 0.4 W/cm².


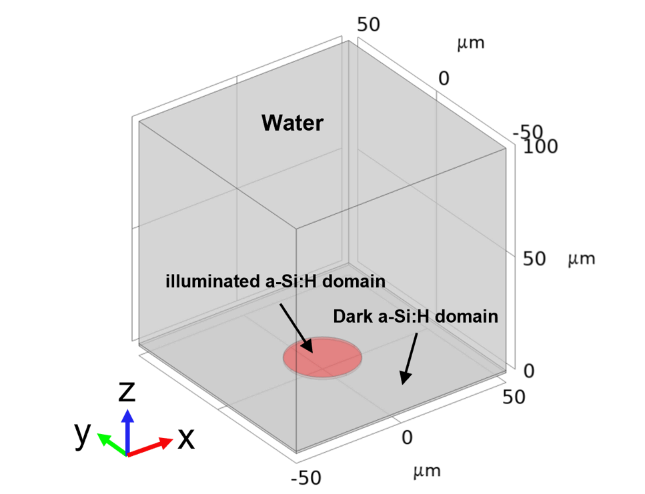


**Figure S2.** Schematic of the 3D simulation model of the OET chip. In the main text, the XZ plane (y=0) was used for the simulation results shown in Figure 2.


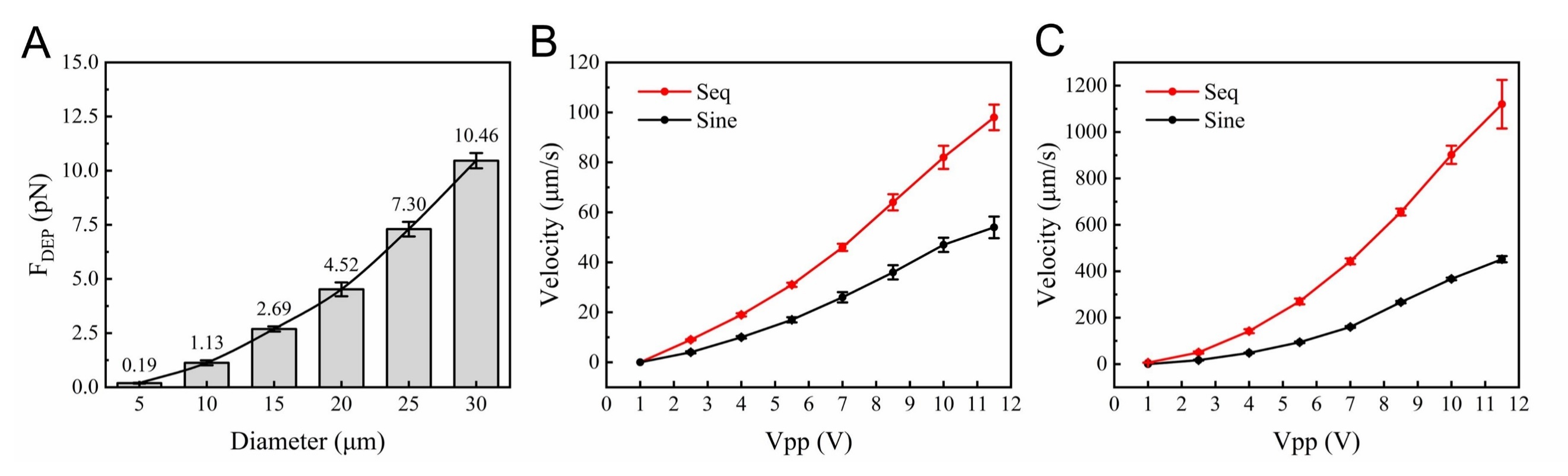


**Figure S3.** Performance of the OET system in manipulating the SiO_2_ and Ag-SiO_2_ microspheres under different conditions. (A) Calculated DEP force in Figure 2J based on the Stokes drag formula, showing that the force increased with the square of the particle diameter. (B) and (C) Manipulating velocity as a function of voltage for SiO_2_ and Ag-SiO_2_ microspheres with a diameter of 20 μm under 50 kHz. The red line represents the rectangular waveform and the black line represents the sinusoidal waveform. The manipulating velocity for the rectangular waveform was approximately twice that for the sinusoidal waveform.


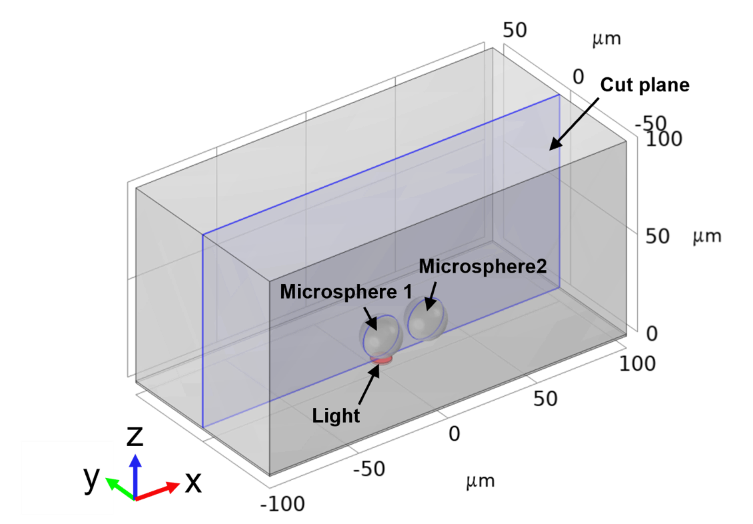


**Figure S4.** Schematic of the 3D simulation model of the two microspheres. In the main text, the XZ plane (y=0) was used for the simulation in Figure 5.


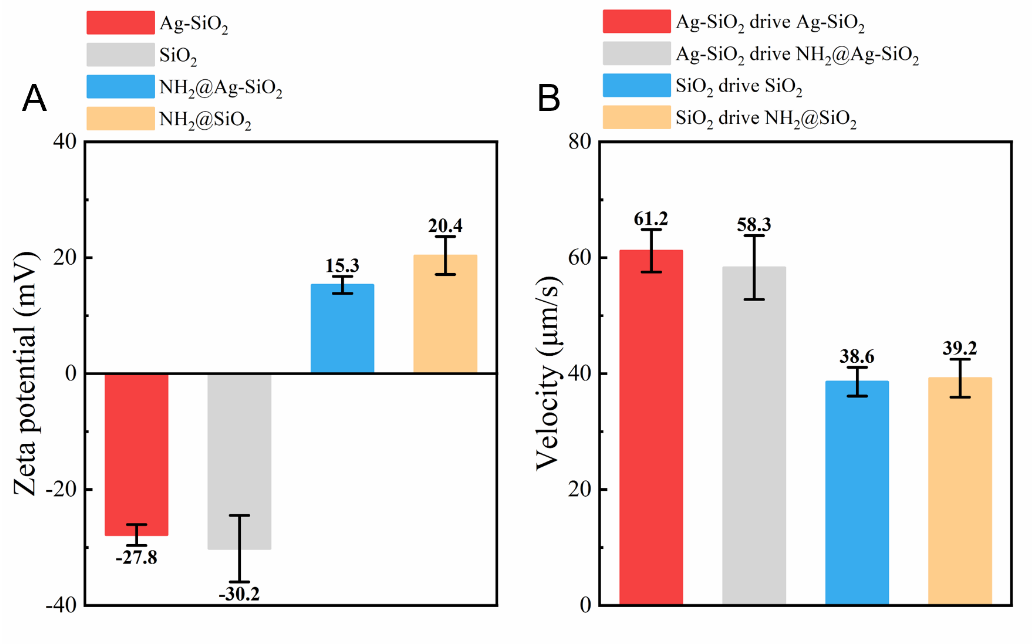


**Figure S5.** (A) Zeta potentials of Ag-SiO_2_ and SiO_2_ microspheres before and after amination. (B) Comparison of particle-assisted OET manipulation speeds for microspheres with different zeta potentials.


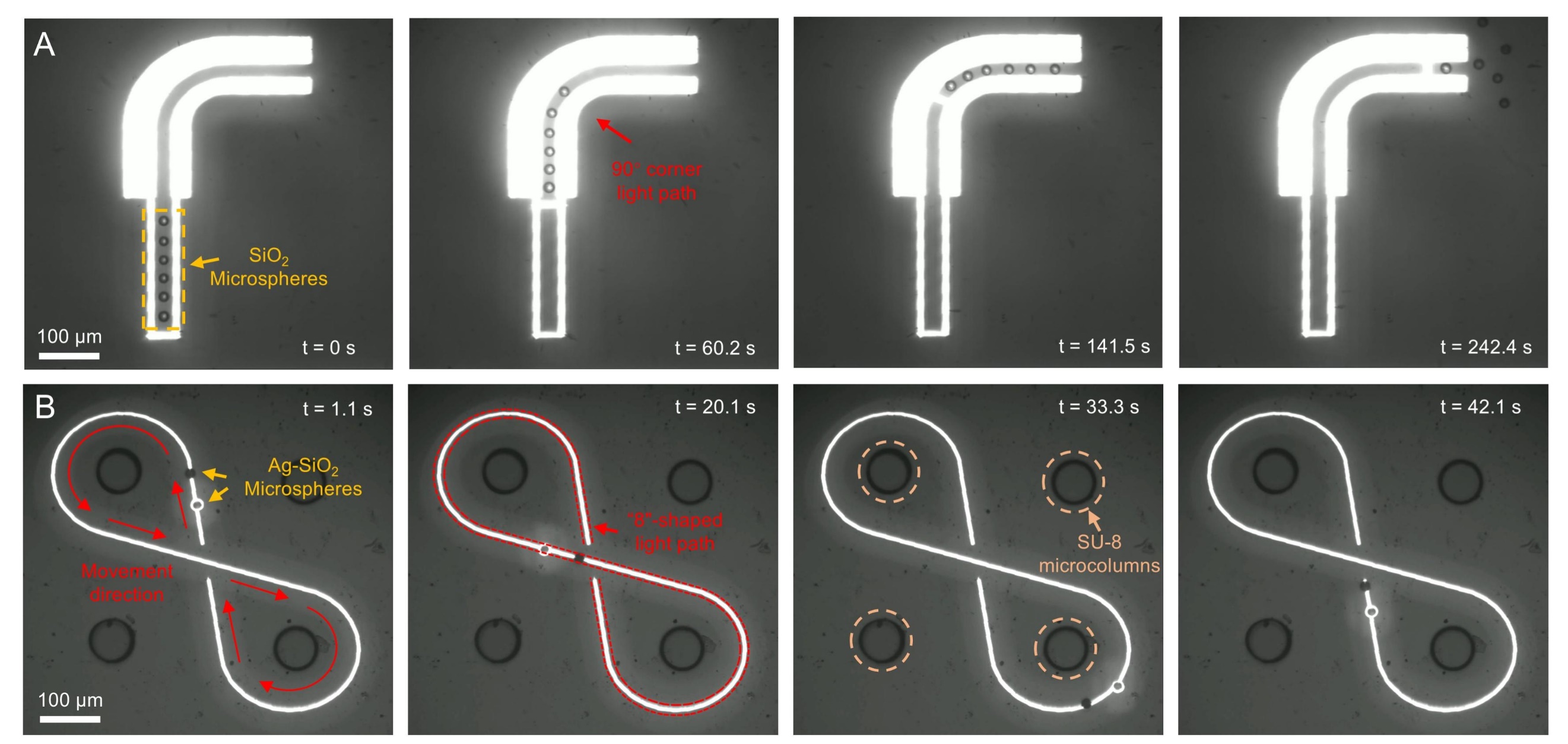


**Figure S6.** Particle-assisted OET manipulation of the SiO_2_ and Ag-SiO_2_ microspheres. (A) Particle-assisted OET drives multiple SiO_2_ microspheres along a light path with a 90° corner, demonstrating that PiDEP can transfer multiple microspheres. (B) Transportation of an Ag-SiO_2_ microsphere around obstacles along an "8"-shaped light path using particle-assisted OET. The obstacles are microcolumns fabricated from the SU-8 photoresist.





**Figure S7.** Maximum driving velocity of 293T cells manipulated by microspheres with different metal coatings.


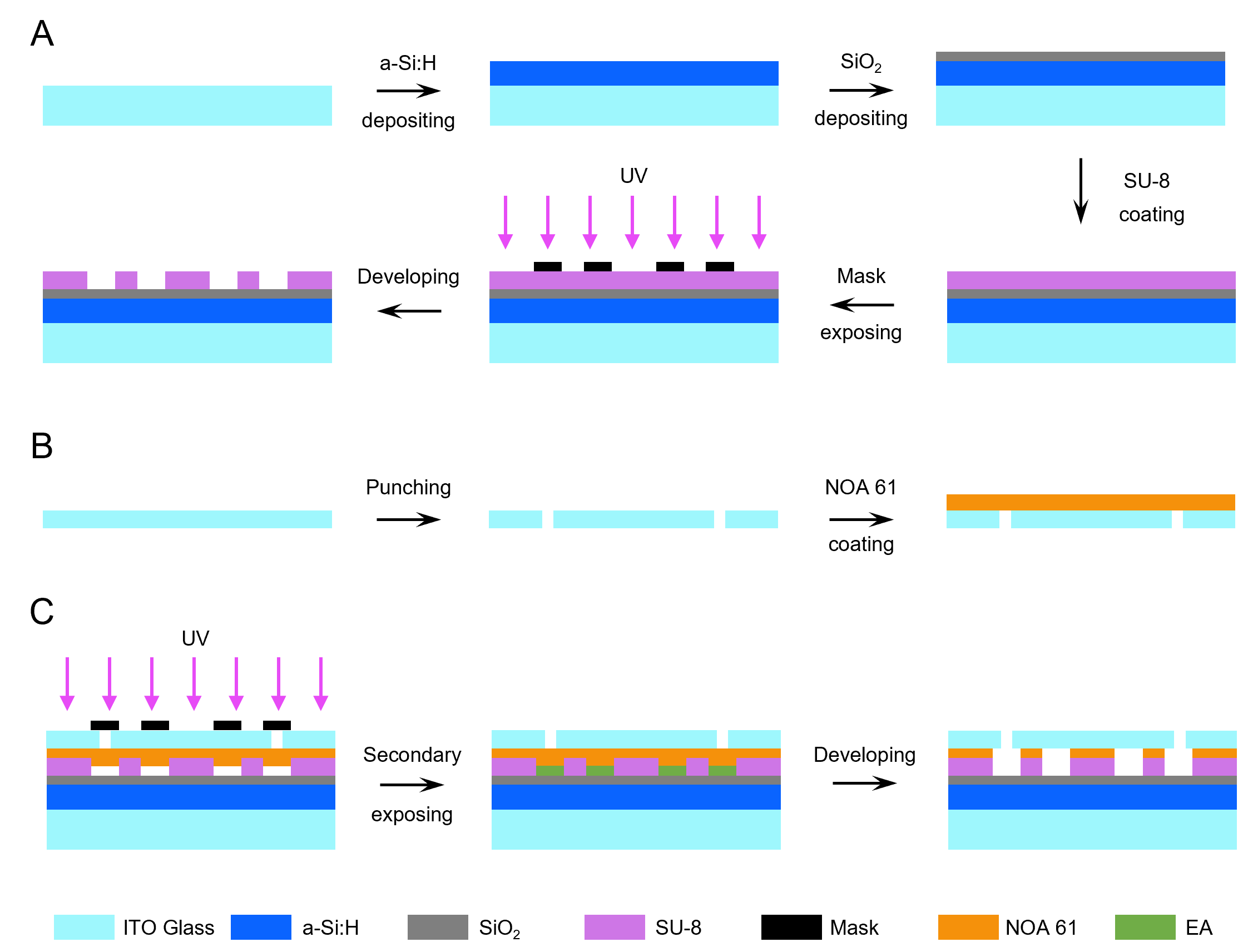


**Figure S8.** Fabrication process of the OET chip based on in-situ photolithography. (A) Preparation of the OET chip substrate. (B) Preparation of the OET chip cover plate. (C) Assembly and packaging of the OET chip. The detailed steps followed in the preparation processes are provided in the main text.

| Parameter | Description | Value |
| --- | --- | --- |
| $\sigma_{SiO_{2}}$ | Conductivity of SiO_2_ | $1\times10^{-9}\text{S}/\text{m}$ |
| $\varepsilon_{SiO_{2}}$ | Electrical permittivity of SiO_2_ | $3.9\varepsilon_{0}$ |
| $\sigma_{Ag}$ | Conductivity of argentum | $6.3\times10^{7}\text{S}/\text{m}$ |
| $\varepsilon_{Ag}$ | Electrical permittivity of argentum | $1\times10^{9}$ |
| $\sigma_{m}$ | Conductivity of medium | $2\times10^{-3}\text{S}/\text{m}$ |
| $\varepsilon_{m}$ | Electrical permittivity of medium | $78.5\varepsilon_{0}$ |
| $R$ | Radius of SiO_2_ | $10\times10^{-6}\text{m}$ |
| $r$ | Radius of Ag-coated SiO_2_ | $10.1\times10^{-6}\text{m}$ |

**Table S1.** Parameters for calculating Re(*K*).

| Parameter | Description | Value |
| --- | --- | --- |
| $\sigma_{cyto}$ | Intracellular conductivity | $0.5\text{ S}/\text{m}$ |
| $\varepsilon_{cyto}$ | Intracellular electrical permittivity | $50\varepsilon_{0}$ |
| $\sigma_{mem}$ | Cell membrane conductivity | $3\times10^{-6}\text{ S}/\text{m}$ |
| $\varepsilon_{mem}$ | Cell membrane electrical permittivity | $8\varepsilon_{0}$ |
| $d_{mem}$ | Cell membrane thickness | $10\times10^{-9}\text{ m}$ |

**Table S2.** Cell parameters used for calculation Calusius-Mossotti factor and simulation

| Parameter | Description | Value |
| --- | --- | --- |
| $\sigma_{m}$ | Conductivity of medium | $2\times10^{-3}\text{S}/\text{m}$ |
| $\varepsilon_{m}$ | Electrical permittivity of medium | $78.5\varepsilon_{0}$ |
| $\sigma_{silicon-light}$ | Conductivity of amorphous silicon in the light | $1\times10^{-4}\text{S}/\text{m}$ |
| $\sigma_{silicon-drak}$ | Conductivity of amorphous silicon in the dark | $1\times10^{-6}\text{S}/\text{m}$ |
| $\varepsilon_{silicon}$ | Electrical permittivity of silicon | $11.7\varepsilon_{0}$ |

**Table S3.** COMSOL parameters for the OET chip simulation.

Supplementary References

[1] G. C. Spalding, K. Dholakia, A. Wheeler, S. Zhang, presented at *Optical Trapping and Optical Micromanipulation XV*, **2018**.

[2] S. Zhang, N. Shakiba, Y. Chen, Y. Zhang, P. Tian, J. Singh, M. D. Chamberlain, M. Satkauskas, A. G. Flood, N. P. Kherani, S. Yu, P. W. Zandstra, A. R. Wheeler, *Small* **2018**, *14* (45), e1803342, https://doi.org/10.1002/smll.201803342.

[3] S. Liang, C. Gan, Y. Dai, C. Zhang, X. Bai, S. Zhang, A. R. Wheeler, H. Chen, L. Feng, *Lab Chip* **2021**, *21* (22), 4379, https://doi.org/10.1039/d1lc00610j.
